# Supplementary material for: Patterns of Intron Gain and Loss in Fungi
Source: PLoS Biol. 2004 Nov 30;2(12):e422. doi: 10.1371/journal.pbio.0020422 (PMC532390; doi:10.1371/journal.pbio.0020422)
Supplement: Table S1 — Also available at http://genes.mit.edu/NielsenEtAl/. (4.3 MB ZIP). [file pbio.0020422.st001.zip › NielsenEtAl/html/1318.html]

AN6522.1.NCU04149.1.MG04070.1.FG05220.1


```
 CLUSTAL W (1.82) Multiple Sequence Alignments - Introns Inserted


Sequence 1: NCU04149.1	530 aa
Sequence 2: FG05220.1	436 aa
Sequence 3: MG04070.1	535 aa
Sequence 4: AN6522.1	614 aa
Alignment Length: 648 aa
Number Identitical Residues: 176 aa
Alignment Score (without introns) 9161


MG04070.1 	------------------------------------------------------------
NCU04149.1	------------------------------------------------------------
FG05220.1 	------------------------------------------------------------
AN6522.1  	MRASPFWHLRRVATPVRLPRNQPWAKAHPLCHALNTSVSSTPQHRCFAATVPSRLLYKGD
          	  ::.       ::.   . ... :.: . . : .:: :::..  . ::: .:    ...

MG04070.1 	------------MIQFTHSLPHKEEP0-~IYRRLCPGKKPPKPRYAAVRTLYAP------
NCU04149.1	------------MSLAVGRLPLTPSL~G0VYNSLCPTKLIPKPRYAAVRTLTEPTEPGND
FG05220.1 	--------------------------~-~-------------------------------
AN6522.1  	STIYALSTAPGRAAIAVVRVSGPACV~S0IYQALCPKAPLPRPRVAAVRTIYDP------
          	::  : :::..         .      .      ..    . .  ::  :   .      

MG04070.1 	---ENRPGTPDVLDSGALVLYFPGPKTVTGE~DVLELHTHGGRATVKSVLSAIPQCAPAA
NCU04149.1	VSSPSAANANNVLDTDALVLYFPGPKTVTGE~DVLELHVHGGPATVKAVLSAIPKST-SS
FG05220.1 	---------------------------MSRD1DVLELHVHGGSATVKAVLAAIPKCA-TA
AN6522.1  	-ASDEPCGQADILDAGALVLYMPGPKTATGE~DVLEFHLHGGPAIVKSVLSAISKTSGKE
          	 :: .  .  .  .:.:     ....: : : ****:* *** * **:**:**.: :   

MG04070.1 	SRVRYADPGEFTRRAFFNDRLDLAQVESLGDALDAETEQQRRAAVRGSSGRLGASYDNWR
NCU04149.1	STIRYAEQGEFTKRAFLNNRLDLAQVEALGDTLSAETEQQRRAAIRGTSGVLGKTYESWR
FG05220.1 	HRIRYAEPGEFTKRAFFNNRLDLAQIESLSDTLAAETEQQRRAAVRGNSGSLGRQYEAWR
AN6522.1  	HSVRYAEPGEFTRRAFMNDRLELPQIEALGDTLAADTEQQRRLAVRGASDALSKRYEKWR
          	  :***: ****:***:*:**:*.*:*:*.*:* *:****** *:** *. *.  *: **

MG04070.1 	ERLLLARAEIEALIDFSEDQHFDESPRELLQSVVGQVRDILALIALHEMGSKRSELLRKG
NCU04149.1	EQLLLARGEIEALIDFSEDQHFDESPTELLRNVTRLVKEILHSIKLHELGSQRSELLRNG
FG05220.1 	EQLLLTRGEIEALIDFSEDQHFDESQAELLQNVTAQVARMLHSIELHEQGSQRSELLRNG
AN6522.1  	QQLLYARGELEALIDFSEDQYFDESSDDLVLSVSTQVRALSRQIALHIQNASKGELMRSG
          	::** :*.*:**********:****  :*: .*   *  :   * **  .:.:.**:*.*

MG04070.1 	IRIALLGPPNVGKSSLMNLIVGREASIVSTEAGTTRDIVEASLDIRGYLCTFADTAGFRS
NCU04149.1	IRIALLGPPNVGKSSLMNLIVGREASIVSSEAGTTRDIVEASLDIRGYLCSFADTAGIRT
FG05220.1 	IRIALLGPPNVGKSSLMNLIVGREASIVSGEAGTTRDIVEASLDIRGYLCSFADTAGFRS
AN6522.1  	IKVALLGAPNAGKSSLLNRIVGREAAIVSTEEGTTRDIVDVGIDLGGWYCKLGDMAGIRS
          	*::****.**.*****:* ******:*** * *******:..:*: *: *.:.* **:*:

MG04070.1 	EET--TDASSPAEIGIVEKEGIRRARSKAIGSDIVVVLASVEKQGNG--EYSICYEQETV
NCU04149.1	RSSLLPAEEKEASIGKIEEEGIRRARQKALDSDVIIVLASVEPHPNGVEGYRLNYDLSTL
FG05220.1 	QSSQITSEAESGAIGAVEEEGIRRAKQRALESDLVIVLASVEKGPR---GFFLQYDEETL
AN6522.1  	EPSSNSPGGASLVIGSVEKEGIRRARARAMASDVVVVVISVEEGSDEGAPFRLAMEPDVV
          	. :  .       ** :*:******: :*: **:::*: ***        : :  : ..:

MG04070.1 	QLAAQAPQS----LVVINKSEQVP---ASALKTLVAGFKARVLAPFS----GLQGAEPLV
NCU04149.1	DLASSAPES----LLVINKSETVP---PSVLAQLIQSFKTDVLSTALPEDSPLRSQDPIL
FG05220.1 	DLAAGAEDC----LVVVNKQDAVD---KVEFGKLVQKFRQNAQLRAP----KLAAAELVS
AN6522.1  	DAANECVRAGKQIIVAINKCDRLPPLDQSEASQVPHEIAQKVNQIFP----EVPKKRVFG
          	: *  .  .... ::.:** : : . .      :   :   .          :     . 

MG04070.1 	ISCKEGGNADAVGLKNNEADPGAIHALVDSLSARFESLTSLPDD--------MQDLLGVT
NCU04149.1	ISCR---TAEQRSGGVDDTDPGKIHHLISRLSSSFAELTAIPQD--------MEHLLGVT
FG05220.1 	ISCK---EAQ--TGSWESKDPGGIQAVITRLVESFERMTSMPVD--------LQDLLGVT
AN6522.1  	ISCLE--SSDGLEQPMTNSWPSFLQGLISTFEEMATPAGVDGDANGQYDRSYWEDSLGVS
          	*** .   ::       .  *. :: ::  :             ... . :  :. ***:

MG04070.1 	ERQGQLLARCRGHLDDFISEATRSSDEPGSQ--------IYQHEEDIDVVLAAEHLRYAA
NCU04149.1	ARQNELLGQCRDALEDFMAEANPNPNPAIGEG------GITGEEVEADIVLAAEHLRVAA
FG05220.1 	ERQRQLLIKCRQHLEDFMVEATPE------------------EGMDADTVLAAEYLRYAA
AN6522.1  	HRQSSNLQLCLQSLEEFLAQANPSSSRDTPDGPYSGSPGPDSVESEVDIVVAAEHLRYAA
          	 ** . *  *   *::*: :*. ...    ... :.:..      : * *:***:** **

MG04070.1 	NALAEITGRGGYGDTEEVLGVIFEK2FCVG1ERDRGSDAPGPFPYIPGYFLPR
NCU04149.1	GRLAAITGKGEAGDVEEVLGVIFEK~FCVG~K---------------------
FG05220.1 	DCLARITGRGEFGDVEDVLGVIFEK2FCVG~K---------------------
AN6522.1  	DALASITGKGESGDVEDVLGVVFEK~FCVG~K---------------------
          	. ** ***:*  **.*:****:*** **** :
```
